# PVA/Inulin-Based Sustainable Films Reinforced with Pickering Emulsion of Niaouli Essential Oil for Potential Wound Healing Applications

**DOI:** 10.3390/polym15041002

**Published:** 2023-02-17

**Authors:** Fatma Nur Parın, Sofia El-Ghazali, Ayşenur Yeşilyurt, Uğur Parın, Azeem Ullah, Muzamil Khatri, Ick Soo Kim

**Affiliations:** 1 Department of Polymer Materials Engineering, Faculty of Engineering and Natural Sciences, Bursa Technical University, Bursa 16310, Turkey; 2 Nano Fusion Technology Research Group, Institute for Fiber Engineering (IFES), Interdisciplinary Cluster for Cutting Edge Research (ICCER), Shinshu University, Tokida 3-15-1, Ueda 386-8567, Japan; 3 Central Research Laboratory, Bursa Technical University, Bursa 16310, Turkey; 4 Department of Microbiology, Faculty of Veterinary Medicine, Aydın Adnan Menderes University, Aydın 09100, Turkey; 5 Department of Chemistry and Materials, Shinshu University, Ueda 386-8567, Japan

**Keywords:** PVA/inulin, Pickering emulsions, pumpkin powder, niaouli essential oil, sustainable film

## Abstract

In this study, sustainable water-based films were produced via the solvent-casting method. Petroleum-free-based polyvinyl alcohol (PVA) and carbohydrate-based inulin (INL) were used as matrices. Vegetable-waste pumpkin powder was used in the study because of its sustainability and antibacterial properties. Pickering emulsions were prepared using β-cyclodextrin. The influence of the different ratios of the β-cyclodextrin/niaouli essential oil (β-CD/NEO) inclusion complex (such as 1:1, 1:3, and 1:5) on the morphological (SEM), thermal (TGA), physical (FT-IR), wettability (contact angle), and mechanical (tensile test) characteristics of PVA/inulin films were investigated. Moreover, the antibacterial activities against the *Gram* (*−*) (*Escherichia coli* and *Pseudomonas aeruginosa*) and *Gram* (*+*) (*Staphylococcus aureus*) bacteria of the obtained films were studied. From the morphological analysis, good emulsion stability and porosity were obtained in the Pickering films with the highest oil content, while instability was observed in the Pickering films with the lowest concentration of oil content. Thermal and spectroscopic analysis indicated there was no significant difference between the Pickering emulsion films and neat films. With the addition of Pickering emulsions, the tensile stress values decreased from 7.3 ± 1.9 MPa to 3.3 ± 0.2. According to the antibacterial efficiency results, films containing pumpkin powder and Pickering emulsion films containing both pumpkin powder and a ratio of 1:1 (β-CD/NEO) did not have an antibacterial effect, while Pickering emulsion films with a ratio of (β-CD/NEO) 1:3 and 1:5 showed an antibacterial effect against *Escherichia coli*, with a zone diameter of 12 cm and 17 cm, respectively. Among the samples, the films with ratio of (β-CD/NEO) 1:5 had the highest antioxidant capacity, as assessed by DPPH radical scavenging at 12 h intervals. Further, none of the samples showed any cytotoxic effects the according to LDH and WST-1 cytotoxicity analysis for the NIH3T3 cell line. Ultimately, it is expected that these films are completely bio-based and may be potential candidates for use in wound healing applications.

## 1. Introduction

The demand for products such as polymer films has been growing steadily, and plastic waste is estimated to reach 33 billion tons by 2050. As a result, considerable effort has been invested to decrease polymer film waste [1,2]. Biodegradable films have gained a lot of attention in recent times because of the serious safety issues caused by the hard degradation of petroleum-based plastic materials [3,4,5]. Natural biopolymers (alginate, chitosan, gelatin, cellulose, inulin, and starch), synthetic biodegradable polymers polyvinyl alcohol (PVA), polylactic acid (PLA), and polycaprolactone (PCL)) were used to obtain biodegradable films [3,6,7,8]. The advancement of biodegradable films for the medical, cosmetic, and pharmaceutical fields seeks to enhance functional characteristics, such as drug delivery, and mechanical and safety concerns. Amongst the biodegradable polymers, poly(vinyl alcohol) (PVA) has become one of the most widely utilized in many applications based on its optical stability, biocompatibility, non-toxicity, and good mechanical property [3]. Despite PVA’s unique properties, some of these properties must be modified or enhanced to extend their potential usage [9,10]. Therefore, the preparation of a composite film by adding functional biomaterials and obtaining a blend film could be a potential strategy to overcome the drawback of PVA being a synthetic polymer [11].

Among the natural polymers mentioned above, inulin (INL), a biomaterial found in abundance in Jerusalem artichoke, can be used as a new biodegradable biomaterial [12,13]. INL is a type of storage polysaccharide found in the colloidal form in the rhizomes of chrysanthemums, lilies, and other plants that are moderately water-soluble. INL is a type of prebiotic [14]. INL has found numerous medical applications: in controlling cholesterol level, weight loss, and constipation, and good functioning of the urinary tract. In the food industry, it is utilized as a substitute for sugar in diabetic patients [15]. The associated properties, namely, biodegradability, biocompatibility, non-toxicity, antibacterial, and hydrophilicity, promote it as a suitable material for biomedical applications [15,16]. INL is reported to improve wound healing by regulating oxidative and inflammatory responses. Topical application of INL enhances keratinocyte migration, accelerates re-epithelialization, and increases the fibroblast reaction on skin wounds [17]. INL’s physicochemical properties, such as its film-forming potential, allow it to be combined with other polymers to produce blend films [18]. Therefore, INL was selected for the preparation of a blend solution with PVA.

There are some difficulties in producing polymer films containing essential oils, due to essential oils’ instability and sensitivity to external conditions such as temperature, light, and even oxygen [19,20]. Meanwhile, essential oils are beneficial, but their direct use is restricted due to their high volatility and their low solubility in water [3]. Preparing a highly stable oil/water (*o*/*w*) emulsion is one effective method of solving these problems [21]. Pickering emulsions are identified as a more effective and sustainable alternative to surfactant-stabilized (traditional) emulsions [22]. Pickering emulsifiers are defined as solid particles that collect at oil–water interfaces They are derived from nature and are a nontoxic substitute for surfactants in emulsion formulations [23]. Pickering emulsions based on polysaccharides have gained popularity in the last 10 years [23]. Even though inorganic particles such as silica, clay, and titanium dioxide have been used as Pickering emulsifiers, other studies have used modified cellulose, protein, and starch to improve biocompatibility. Thus, Fasihi et al. (2019) synthesized PVA/CMC Pickering films for active food packaging [24]. In another study, Zhou et al. (2018) prepared oregano essential oil (OEO) Pickering emulsions using cellulose nanocrystals (CNCs) for the food industry and evaluated the antimicrobial properties [25]. Pickering pectin films stabilized with marjoram essential oil were synthesized by Almasi and Amjadi (2020) for potential food packaging [26].

Cyclic oligosaccharide beta-cyclodextrin (β-CD) is obtained from the controlled enzymatic hydrolysis of starch and is formed by seven D-glucopyranose units linked by 1,4-glucosidic bonds [27,28]. β-cyclodextrin (β-CD) with a hydrophobic cavity interior and hydrophilic cavity exterior structure acts as a very good Pickering emulsifier at room temperature due to both the solubility in water and the capability of the encapsulation of the hydrophobic ingredients [28]. Recently, Parın (2023) produced β-cyclodextrin-stabilized, cinnamon essential-oil-loaded PVA/egg white foams for wound dressing applications and achieved good antimicrobial results [29].

It is reported that a substantial amount of the literature focuses on the fabrication and characterization of essential-oil-loaded polymer films. Nevertheless, no studies have been conducted on essential oil Pickering emulsions stabilized with β-cyclodextrin nanoparticles that are miscible with a PVA/inulin matrix to produce polymer films as bio-based materials. However, biowaste pumpkin powder was used both to develop material properties and for sustainability. Thus, this study intended to fill this knowledge gap. The morphological, thermal, physical, mechanical, and antibacterial properties of the film were also studied based on the changing essential oil amount.

## 2. Materials and Methods

### 2.1. Materials

PVA (Polyvinyl alcohol) powders (30,000 Mw, purity 95%, degree of hydrolysis 87.8%) and inulin (INL) powders (average DP is 20–22) were purchased from Zag Chemical Company, Turkey, and Vegrano, Turkey, respectively. β-cyclodextrin was kindly provided by Wacker Chemical Company, Germany. Citric acid was acquired from Aksu, Turkey. MBAm (*N*, *N*′-Methylenebis(acrylamide) (99% purity) was supplied from Sigma-Aldrich St. Louis, MO, USA. Ammonium persulfate (APS) ((NH_4_)_2_S_2_O_8_) (initiator, 98%, Sigma-Aldrich, St. Louis, MO, USA) (Potassium persulfate (KPS) (K_2_S_2_O_8_) (initiator, 98%, Merck, Darmstadt, Germany) was used as received. All the chemicals were used in the experiments without being purified.

### 2.2. Fabrication of PVA/Inulin Films

A homogenous 10% (*w*/*v*) PVA solution was obtained using distilled water at 90 °C. Then, 1.5 g of inulin powders were added to the PVA solution, and the polymer blend solution was mixed overnight. MBAm (crosslinker) was added to the mixture, after glycerol and citric acid. The initiator’s KPS and APS (a certain amount) were added to the blend solution. This neat polymeric solution was put into a Teflon mold and cured at 70 °C overnight. In this study, citric acid was used as a physical crosslinker. Glycerol was selected as a plasticizer, which is a commonly used substance in the food industry [30].

**Figure 1:**
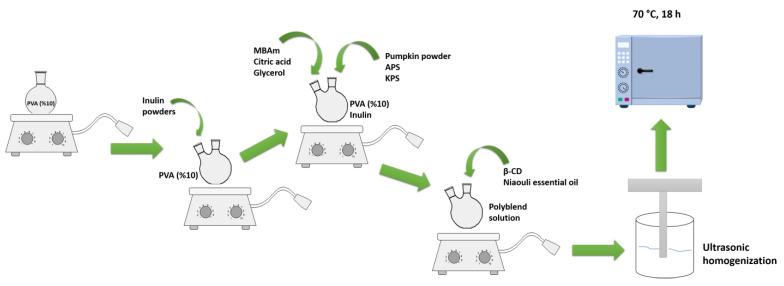
Schematic illustration of Pickering films.

**Figure 2:**
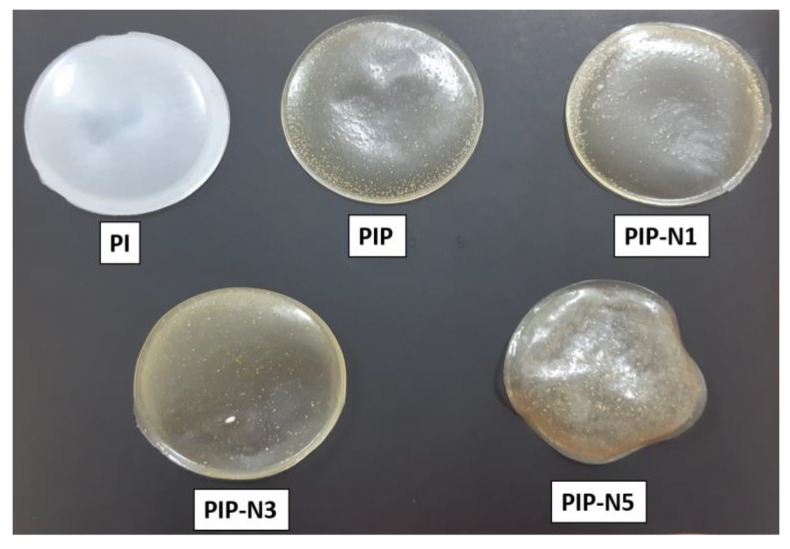
Digital images of the film samples.

To obtain polymer films with pumpkin powders, the same procedure was carried out. Thus, pumpkin powder was added before the addition of initiators. On the other hand, to produce Pickering film, β-cyclodextrin was added to the polymer solution and mixed in a high-speed magnetic mixer. After the mixture became homogeneous, niaouli essential oil was added to the polymer solution drop by drop. As in other experiments, pumpkin powder was added to the system last and left to mix overnight. Figure 1 provides a schematic for films preparation. The compositions of polymer films are given in Table 1. Figure 2 shows digital images of all film samples.

### 2.3. Characterization of Films

The morphology of the produced films was evaluated using a Carl Zeiss/Gemini 300 Scanning Electron Microscope (SEM), Ankara, Turkey. All samples were gold-coated before analysis. The non-conductive polymer film samples were cut into a shape of 1 cm × 1 cm and placed in the stub. The surface of the samples was coated with Au/Pd (60/40) by the chemical evaporation deposition (CVD) method.

A stereomicroscope was used to examine the exterior structures of the neat and produced films (Leica-BM 2500M).

The chemical groups of the neat and niaouli oil-added films were confirmed using Fourier-transform infrared (FTIR) spectroscopy. The data were collected using a Thermo Nicolet iS50 FT-IR (Waltham, MA, USA) spectrometer with an ATR adaptor (Smart Orbit Diamond, Brooklyn, WI, USA) in the wavelength range 4000–500 cm^−1^, with 16 scans at 4 cm^−1^ resolution.

TGA was performed in a nitrogen atmosphere (N_2_) with a heating rate of 10 °C min^−1^ over a temperature range of 30–600 °C, followed by an oxygen atmosphere (O_2_) with the same heating rate over a temperature range of 600–900 °C.

The wettability test of the resultant films was investigated via Attention Theta Lite optical contact angle goniometer (Biolin Scientific, Gothenburg, Sweden), and 4 µL distilled water samples were dropped by 2 cm × 2 cm. For each sample, at least three replications were performed.

The tensile strength and elongation at break of all film samples were determined using an ASTM D882-compliant universal testing machine (Testform/AS1, Ankara, Turkey) with a crosshead speed of 10 mm/min and a 1 kN load cell. The mechanical performance of each sample was evaluated using five separate measurements, and the average data were calculated with standard deviations. Mechanical tests on all samples were carried out at 50% relative humidity and room temperature to ensure the same moisture content.

### 2.4. Antibacterial Activity Measurements

Staphylococcus aureus ATCC^®^ 25923, Escherichia coli ATCC^®^ 25922, and Pseudomonas aeruginosa ATCC^®^ 15692 standard bacterial strains were used as positive controls in the study. Bacterial strains were grown on solid media. Petri plates were incubated for 24 h at 37 °C in under aerobic environment [31].

Then, 100 µL of broth inoculum pre-adjusted at 0.5 McFarland turbidity standard with the bacterial strains (1 × 10^8^ CFU/mL) was streaked on the surface of Mueller Hinton agar plates. The polymer films (5 mm dia.) were placed on the agar surface. Then, Petri plates were incubated at 37 °C for 24 h under an aerobic environment. At the end of incubation, inhibition zone diameters around each polymer disc were qualitatively measured with a caliper (CLSI, 2017).

At the end of the incubation of the Petri plates, all samples in the *Staphylococcus aureus* ATCC^®^ 25923, *Escherichia coli* ATCC^®^ 25922, and *Pseudomonas aeruginosa* ATCC^®^ 15692 Petri plates and No:5 polymer in the *Escherichia coli* ATCC^®^ 25922 Petri plates were determined for inhibition zone diameters.

### 2.5. DPPH Free Radical Scavenging

For measuring the antioxidant activity of the produced films, 20 mg of the film sample was immersed in 100 μL of DPPH solution in ethanol and 80 μL of assay buffer. The samples carrying solutions were incubated for time intervals of 2 h, 6 h, and 12 h. At specific time intervals, the absorbance at 517 nm was measured using a UV–Vis spectrophotometer (Lambda 900, PerkinElmer, Waltham, MA, USA). The antioxidant activity was reported as a percentage using the following equation.

(1) $$Antioxidant\;activity\;\left(\%\right)=\frac{Acontrol-Asample}{Acontrol}\times 100\;$$

where *A_sample_* and *A_control_* are the absorptions of DPPH with and without nanofiber sheets, respectively.

### 2.6. Biocompatibility Study

Toxicity and cell viability were conducted by measuring lactase dehydrogenase (LDH) release and WST-1 assay, respectively. Briefly, NIH3T3 cells were cultured at 10^4^ cells per well in a 96-well glass culture plate that had DMEM accompanied with 10% FBS for 24 h. The prepared films were cut into circular discs of 5 mm diameter and were UV-sterilized for 2 h. After sterilization, the films were placed in the wells of 96-well culture plates that had layers of the cultured NIH3T3 cells. The cells were cultured for another 24 h in contact with the films at 37 °C and under a 5% CO_2_ atmosphere. LDH and WST-1 kits were used, in accordance with the protocol of the manufacturer, to measure cytotoxicity and cell viability. The culture medium absorbance for LDH and cell viability was measured at 490 nm and 450 nm, respectively. LDH toxicity was calculated by the following equation.

(2) $$Cytotoxicity\;\left(\%\right)=\frac{A-C}{B-C}\times 100\;$$

where *A* represents the test substance, *B* represents the highly toxic control (lysis buffer), and *C* represents the low toxic control (tissue culture plate).

Cell proliferation of NIH3T3 cells cultured with the prepared film was determined by WST-1 assay on day 1, day 3, and day 7. NIH3T3 cells were cultured using DMEM with 10% FBS at 37 °C under a 5% CO_2_ atmosphere. The treatment medium was replaced every other day. On each predetermined day, WST-1 was added to the culture plate and further incubated for 4 h. Finally, the medium was transferred to a new 96-well plate, the absorbance of the medium was measured at 450 nm by SPECTROstar ^®^ nano microplate reader, BMG Labtech, Offenburg, Germany. For LDH toxicity, cell viability, and cell proliferation, the sample size was 3 (n = 3).

### 2.7. Statistical Analysis

Analysis of variance (ANOVA) and statistical significance (*p* < 0.05) were determined. All statistical analysis was performed using Minitab 21^®^, version 21.2.0, statistical software.

## 3. Results and Discussion

### 3.1. Morphological Analysis

**Figure 3:**
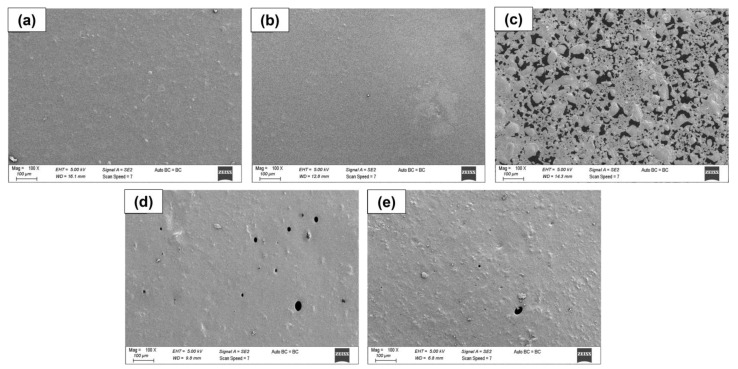
SEM images of the (**a**) PI film, (**b**) PIP film, (**c**) PIP-N1 Pickering film, (**d**) PIP-N3 Pickering film, and (**e**) PIP-N5 Pickering film.

**Figure 4:**
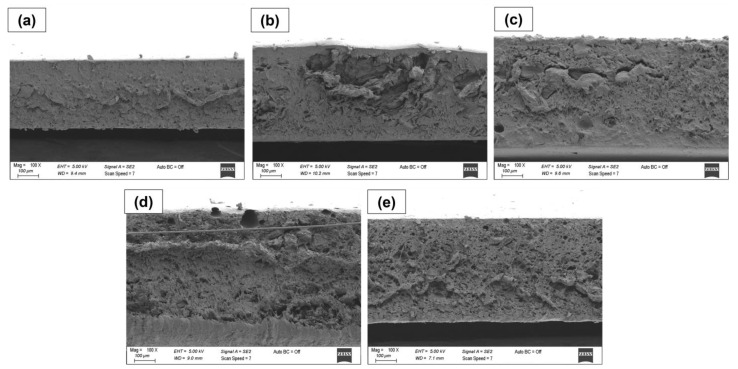
Cross-sectional SEM images of the (**a**) PI film, (**b**) PIP film, (**c**) PIP-N1 Pickering film, (**d**) PIP-N3 Pickering film, and (**e**) PIP-N5 Pickering film.

The surface and cross-sectional images of all materials were analyzed by SEM (Figure 3 and Figure 4). The PVA/inulin (PI) film’s surface and cross-section micrographs (Figure 3) were not entirely smooth and flat and had some pores. However, the blend was mixed by not forming any phase separation, except for the PIP-N1 sample. The concentration of β-CD/essential oil in the aqueous mixture and the niaouli oil/water ratio were the most important factors in the preparation of Pickering emulsions. The SEM images of the emulsion films revealed the production of partially heterogeneous films, particularly for the least amount of niaouli oil (β-CD/niaouli oil ratio of 1:1). The introduction of niaouli essential oil inside the PVA/inulin matrix, on the other hand, resulted in emulsion films with a surface structure attributable to the instability of film-forming emulsions, with indicators such as flocculation and partial phase separation (Figure 3c) [32]. Although there was no change in the surface image with the addition of pumpkin to the polymer solution, it caused a morphological change in the cross-sectional image and an increase in thickness as well. The pumpkin powder was not completely dissolved in the blend solution. Therefore, homogenous films were not obtained. In addition, hollow structures caused pumpkin powder to agglomerate in some regions with the presence of niaouli essential oil. Small porous structures were found on the PIP-N3 and PIP-N5 film surfaces (Figure 4d,e). In the PIP-N3 and PIP-N5 films, which had compact and continuous microstructures, there was no visible separation of oil droplets from the polyblend matrix. As shown in the cross-section images in Figure 4, the PIP-N1 and PIP-N3 films had a decreased value of cross-sectional roughness and cavities (meaning the dispersion of Pickering emulsion droplets was not visible). This is consistent with the literature [33]. When the niaouli oil concentrations increased, smaller pores formed on the surface of the PIP-N5 sample (Figure 3e and Figure 4e). In addition, higher oil concentrations (the PIP-N5 sample) inhibited pumpkin powder aggregation.

### 3.2. FT-IR Spectroscopy

**Figure 5:**
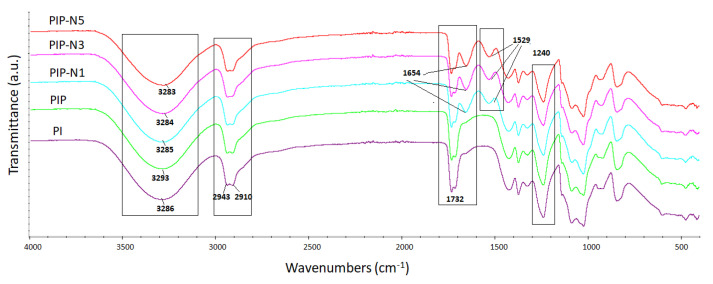
FT-IR spectra of the neat polymer films and sustainable Pickering films.

The functional groups of the polymer films and active compounds (pumpkin powder and niaouli essential oil) were verified using FTIR spectrophotometry, and Figure 5 shows the FTIR spectra of all the film samples.

The spectra of the neat film showed the characteristic peaks of PVA and inulin. As seen in the PI spectrum, the peak at 3293 cm^−1^ corresponded to –OH stretching, whereas the peaks between 2943 (CH group of inulin) and 2910 cm^−1^ corresponded to the asymmetric and symmetric stretching of –CH_2_, respectively. Moreover, the –OH stretching peaks of PVA and inulin overlapped each other. This could be due to hydrogen bonding between the OH groups of both blended materials. The peak at 1732 cm^−1^ could be related to the stretching vibrations of the ester groups (C=O). Furthermore, the geometry of the O–H groups and the configuration of the C–O, C–C, and C–H bonds in the carbon skeleton contributed to the specificity of carbohydrates. The bands from 1500 to 900 cm^−1^ were primarily attributed to C–C and C–O stretching and the C–O–H and C–O–C stretching of some oligosaccharides and polysaccharides. The peaks at 1420, 1373, and 1240 cm^−1^ could be associated with the CH_2_ bending vibration, CH vibration, and OH bending vibration as well [34,35,36,37]. There was a decrease in the peak density of 1732 cm^−1^ with the achievement of the Pickering films. Furthermore, there were also minor changes in the 1100 cm^−1^ peaks.

### 3.3. Thermal Properties

**Figure 6:**
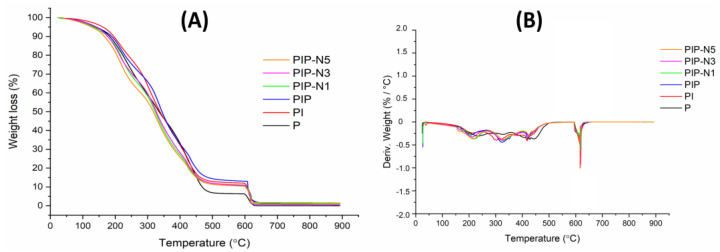
(**A**) TGA curves and (**B**) DTG curves of the neat polymer films and sustainable Pickering films.

The thermal stability patterns of the neat and Pickering films were shown by the TGA curves (Figure 6). All the Pickering and neat films had weight loss at temperatures ranging from 30 to 100 °C. Additionally, three stages of weight loss were observed for all samples. The second weight loss of the PI neat film had five degradation stages. This second stage (165–250 °C) was attributed to the thermal degradation of the bio-blend polymer chains. The ester groups were caused by the impurity of the PVA and ester crosslinks in the inulin chains, according to the literature [38,39,40]. The third stage (250–360 °C) and other sequential weight losses at the range of 360–550 °C were associated with the side chains of the PVA as well as inulin backbone degradation [41]. The final stage was found at 600–650 °C due to the formed carbonaceous residues in the previous stage. The addition of pumpkin powder, surprisingly, lowered the degradation temperatures (Table 2). The TGA curves of the Pickering films were similar to each other. A slight decrease in the thermal stability of the Pickering films, compared to the PI and PIP samples, was observed by adding the oil to the polymer blend solution.

**Figure 7:**
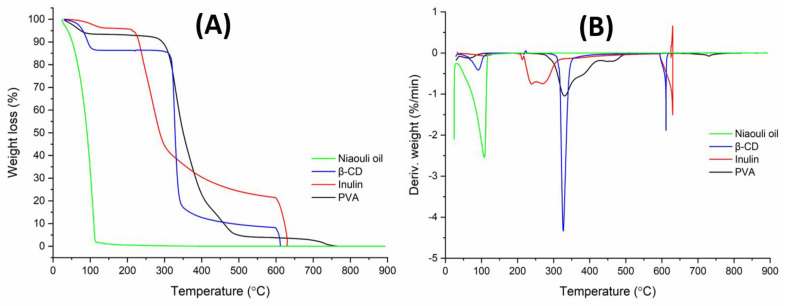
(**A**) TGA curves and (**B**) DTG curves of PVA, inulin, β-CD, and niaouli essential oil.

Figure 7 shows the TGA results for neat PVA, inulin, β-cyclodextrin, and niaouli essential oil. Inside the PVA films, water existed in two states: absorbed water molecules (weak bonds) that prefer to reside on the exterior or interior surface without interacting with the matrix and water molecules that are tightly attached to the –OH groups [42]. The TGA curve of the pure PVA showed three weight degradation, which is consistent with the literature [43]. The loss of absorbed water molecules was assigned to the first stage between 30–150 °C, whereas the loss of water attached to the polymer matrix was attributed to the second stage between 150–360 °C. The third stage (360–500 °C) was related to the breakdown of the polymer main chains [44]. Carbonization occurred after 500 °C.

The curve of inulin revealed three stages of degradation. The first stage (30–150 °C) was due to the moisture amount. The breaking of inulin chains occurred in the second stage (150–320 °C). The last decomposition stage (320–600 °C) could be related to the cellulosic units of the inulins (especially the branching chains of the fructans) and short inulin chains. A third extended decomposition stage between 240 and 320 °C might be associated with the degradation of the branching chains of the fructans (cabuya) [45] and short inulin chains (jicama). The fourth step, which involved a mass loss over 350 °C, might be associated with the entire degradation of the inulin major chains.

β-cyclodextrin exhibited three stages of decomposition. The first stage (30–100 °C) was caused by the loss of absorbed water and crystallization water [46]. The second and third weight losses occurred at 300–600 °C, owing to degradation after the melting of glucose in cyclodextrin [47]. It is important to realize that under the same situations, cellulose is chemically identical to cyclodextrins.

The TGA curve of niaouli essential oil showed one degradation stage. This degradation stage (30–200 °C) might be due to the terpene structures found in niaouli essential oil [48].

### 3.4. Wettability Test

**Figure 8:**
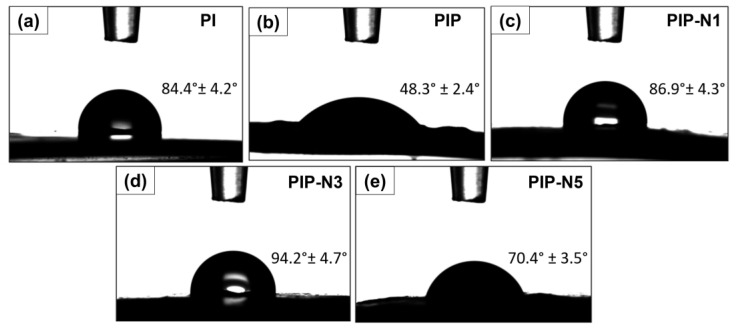
Contact angles of the neat polymer films and sustainable Pickering films.

The water contact angle (WCA) was used to measure the hydrophilicity of materials by capturing the image (Figure 8) [49]. As shown in the WCA values, all samples, except PIP-N3, had a WCA less than 90°, indicating their hydrophilic nature. Figure 8 demonstrates the contact angles of the neat and Pickering films. The PI, PIP, PIP-N1, PIP-N3, and PIP-N5 films had contact angles of 84.4 ± 4, 48.3 ± 2.4, 86.9 ± 4.3, 94.2 ± 4.7, and 70.4 ± 3.5, respectively. The lowest contact angle value of the PIP sample was determined due to the hydrophilic structure of the pumpkin powder [50]. The increment of the hydrophobicity of the films was observed with the preparation of films belonging to the Pickering emulsions, that is, with the addition of oil to the solution. Although the increase in the amount of niaouli oil was high compared to the value of the PIP sample, the contact angle values of the PIP-N5 Pickering film with the highest oil concentration were low. All the Pickering films had a substantially higher WCA value than the PIP films (48.3° ± 2.4). The results confirmed that after containing β-CD-stabilized Pickering emulsions, the PVA/inulin/pumpkin powder films (PIP) had increased hydrophobicity [51]. Increasing the essential oil concentration could cause a rise in the surface hydrophobicity of materials. Nevertheless, the WCA values were influenced not only by the hydrophobicity of the material but also by the surface roughness, surface energy, porosity, and chemical interactions [52]. Despite the increase in the concentration of essential oil, the reason for the decrease in the WCA value might be the presence of more micropores on the surface of the PIP-N5 films (Figure 4e).

Burgos-Diaz et al. (2022) asserted that a stabilizer contact angle lower than 90° is favored for controlling Pickering *o*/*w* emulsions [53]. Moreover, hydrophilic particles (WCA < 90°) are ideal for oil emulsion stability based on the Bancroft rule [54]. Therefore, β-CD particles are hydrophilic, with most of the particles located in the aqueous phase to create an oil-in-water emulsion [55].

**Figure 9:**
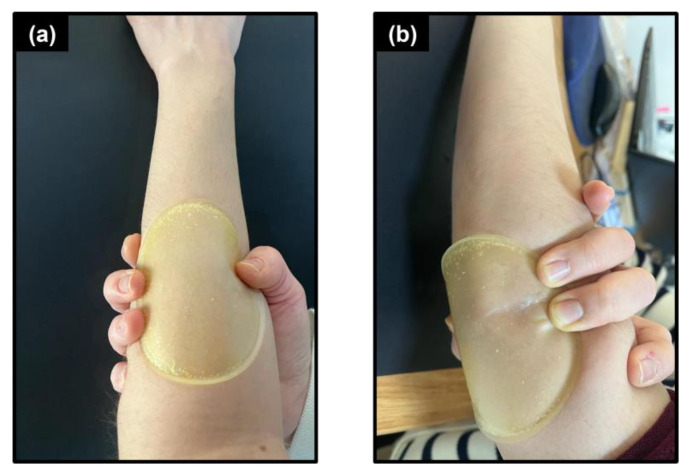
Adhesion of PIP film on (**a**) forearm and (**b**) elbow.

Soft tissue adhesion is an important factor in the selection of materials for wound dressing applications. PVA is known for its excellent film-forming, emulsifying, and adhesion properties. Depending on the type of additives they contain, PVA films can be considered cytocompatible and non-irritating to soft tissue when in contact with them [56]. Figure 9 shows the PIP film attached to the forearm and elbow and that these films require additional supportive layers such as the acrylic adhesion of the soft silicon adhesive for firm attachment on the wound surface.

### 3.5. Mechanical Test

**Figure 10:**
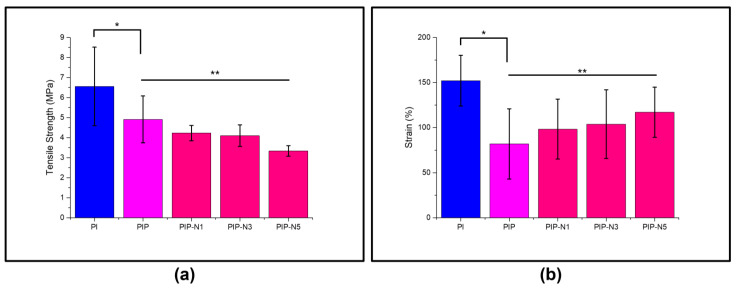
Mechanical performance of prepared films: (**a**) tensile strength (n = 5; mean ± SD, * *p* < 0.0001, *R*^2^ 0.82, ** *p* < 0.0012, *R*^2^ 0.70); (**b**) strain (n = 5; mean ± SD, * *p* < 0.014, R2 0.57, ** *p* < 0.01, *R*^2^ 0.48).

Good mechanical properties are required for a suitable film dressing [57,58]. The effect of the essential oil amount on the elongation at break and tensile strength, for all the film samples, is presented in Figure 10. The tensile strength (TS), and elongation at break (EAB) of the neat, PI, without pumpkin peel, and niaouli essential oil films were found to be 7.3 ± 1.9 MPa and 182.5 ± 5.1%, respectively. The tensile strength and elongation at break values of the film samples decreased when pumpkin peel was introduced to the polyblend (PVA/inulin) solutions. This finding indicated that pumpkin peel caused film hardening. On the other hand, the Pickering films obtained by adding niaouli essential oil to the PVA/inulin/pumpkin peel (PIP) solution had a general increase in the tensile stress and elongation at break values. The essential oils in the polyblend solution showed a plasticizing effect in the Pickering films, increasing the value of elongation, except for the PIP-N5 sample [59].

The Pickering emulsion increased the hydrophobicity of the film matrix, while decreasing the cohesiveness of the polymer matrix, which was especially evidenced by the mechanical properties [33]. There were substantial changes in the TS values for the PIP-N1, PIP-N3, and PIP-N5 films. Furthermore, the Pickering emulsion films demonstrated a steady increase in the EAB values as the amount of niaouli essential oil increased, except for the PIP-N5 sample. This might be linked to the reduced continuous film morphology, because the introduction of niaouli essential oil was achieved by inserting emulsion droplets into the continuous film matrix, which challenged the film’s continuity and cohesion. Moreover, as can be seen from the SEM images, more small pores in the PIP-N5 film also caused both the TS and EAB values to decrease.

Generally, as the amount of niaouli oil increased, the TS value steadily decreased. This could be related to the existence of β-CD/niaouli oil inclusions in the films, which weakened the cohesiveness and resilience of the film. Overall, it is believed that the Pickering films, with PIP-N3 and containing β-CD/niaouli essential oil in a 1:3 ratio mixed better in the polyblend solutions.

### 3.6. Antibacterial Efficiency

**Figure 11:**
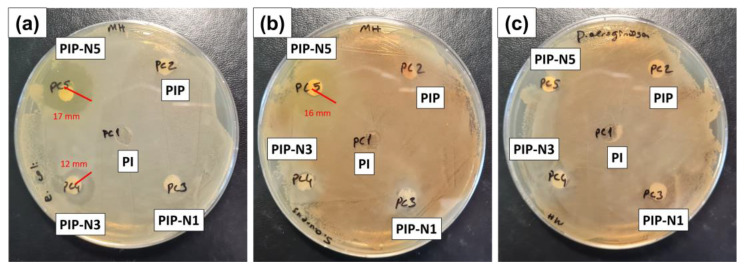
Antibacterial efficiencies of pure PVA/inulin and Pickering films against (**a**) *E. coli*, (**b**) *S. aureus*, and (**c**) *P. aeruginosa*.

A zone inhibition protocol was used to assess the antibacterial efficiencies of the neat and Pickering films against *E. coli*, *S. aureus*, and *P. aeruginosa* (Figure 11).

Bacterial infections have become a major issue for public health as a result of the spread of drug-resistant bacteria, which has enabled the discovery of new antimicrobial therapies [60,61]. In this regard, essential oils are known as “chemical weapons” because their compositions can resist insects or protect plants from bacterial or fungal threats. Essential oils are mainly made up of terpenes and some non-terpene components. Niaouli oil, which is part of the *Myrtaceae* (*Myrtle*) family, consists of 1,8-cineole (oxide) (monoterpene), limonene (monoterpene), a-pinene (monoterpene), ß-pinene (monoterpene), and viridiflorol (sesquiterpene) in varying compositions [62,63]. Table 3 provides the information on the zone of inhibition of the prepared composite films. Fernandes et al. (2022) investigated the effect of niaouli against *Candida fungus*. Their findings indicated that niaouli oil can prevent *C. Auris* planktonic growth, with a 14 mm zone diameter. Furthermore [64], Donoyama and Ichiman (2006) reported that niaouli oil has antibacterial effects on *Staphylococcus aureus*. Niaouli essential oil was effective against pathogenic microorganisms resistant to antibiotics on the skin surface, and the effectiveness of P. aeruginosa on biofilm formation was determined by [65,66]. In another study, Ozdemir et al. (2018) found that niaouli had strong antibacterial resistance of 13.0 ± 1.00 mm, 20.0 ± 1.00 mm, 21.0 ± 1.00 mm, 15.0 ± 1.00 mm, 17.0 ± 1.00 mm, 15.0 ± 1.00 mm, 35.0 ± 0.57 mm, and 13.0 ± 1.00 mm against *Pseudomonas aeruginosa*, *Staphylococcus aureus*, *Escherichia coli*, *Enterococcus faecalis*, *Bacillus subtilis*, *Salmonella typhimurium*, *Staphylococcus epidermidis*, and *Enterococcus hirae*, respectively [67].

In this study, the PIP-N3 and PIP-N5 samples had antibacterial efficiency with *E. coli*. The polymer films obtained by increasing the amount of niaouli oil, that is, by increasing it above a certain value, showed a good antibacterial effect. Gram-negative bacteria are generally more resistant than Gram-positive bacteria. However, contrary to the literature, the PIP-N5 Pickering films showed the highest antibacterial resistance. In addition, only this sample had an antibacterial resistance of 16 mm against *Staphylococcus aureus*.

### 3.7. Antioxidant Activity

**Figure 12:**
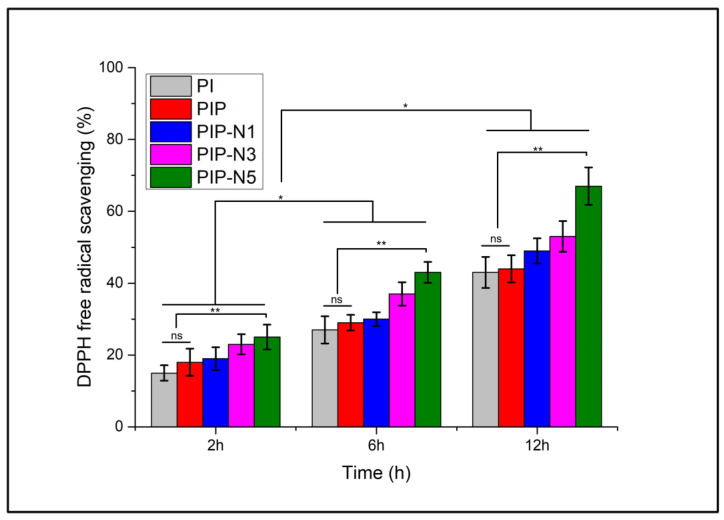
DPPH free radical scavenging activity of produced films (n = 3; mean ± SD, ns: *p* > 0.05 no significant difference, * *p* < 0.001 between time intervals, and ** *p* < 0.0001 against films with no niaouli oil).

Reactive oxygen species (ROS) are commonly produced during respiration, metabolism, or under the stress caused by some diseases and infections. However, a certain amount of ROS is important for maintaining cell homeostasis, though excessive ROS will destroy the antioxidant defense system and break the balance between ROS protection and challenge [68]. Usually, organisms can protect themselves by producing many antioxidants. However, under higher oxidation stress conditions such as injuries, antioxidants are required to be administered. Figure 12 shows the antioxidant activity of the prepared films when coming in contact with the DPPH solution. Film samples without the niaouli oil also showed adequate antioxidant activity, which can be referred to as the inherent antioxidant activity of the inulin and pumpkin powder [69,70]. Niaouli oil-loaded samples showed increased antioxidant activity, with the PIP-N5 (~70%) samples reaching the highest DPPH radical scavenging at 12 h intervals. Furthermore, Figure 12 shows that the antioxidant activity increased as time passes by, which can be justified by the release of niaouli oil from the prepared films into the DPPH solution. Thus, the combination of inulin, pumpkin powder, and niaouli oil served as a good source of antioxidants, if utilized in wound dressing materials.

The PI films showed the least antioxidant effect compared to the other prepared films. The lower antioxidant activity of the PI films was consistent with previous studies, where inulin showed weak antioxidant activity compared to the standard vitamin C [71]. The addition of pumpkin powder slightly improved the antioxidant nature of the PI films, but one-way ANOVA analysis reported no significant differences in the data means, with an R^2^ of 0.0037 and a *p*-value of 0.82. Though pumpkin powder has a strong antioxidant activity due to the presence of linoleic acid and β-carotene, in this research the antioxidant results were not as pronounced as previously reported [72]. The addition of niaouli oil significantly improved the antioxidant activity of the prepared PIP films, with an R^2^ of 0.9209 and a *p*-value of 0.00. With 1,8-cineole being the major component, niaouli oil is a potent antioxidant [73]. The addition of niaouli essential oil improved the antioxidant activity of the composite films.

### 3.8. Biocompatibility Analysis

**Figure 13:**
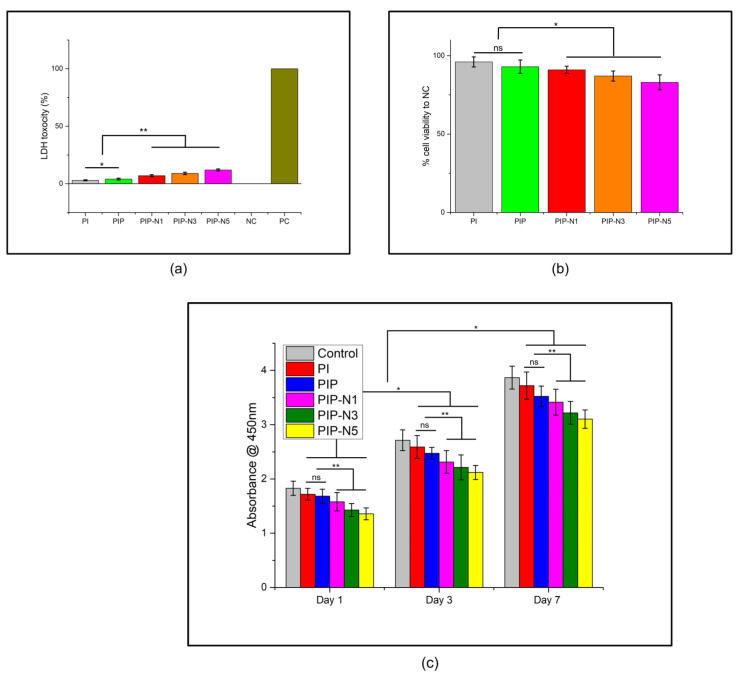
In vitro biocompatibility: (**a**) LDH release assay (n = 3; mean ± SD, * *p* < 0.002, ** *p* < 0.0001), (**b**) cell viability for toxicity (n = 3; mean ± SD, ns: *p* > 0.05, * *p* < 0.001), and (**c**) WST-1 cell proliferation assay (n = 3; mean ± SD, ns: *p* > 0.05, * *p* < 0.0001, ** *p* < 0.013).

Materials that must encounter human skin, especially for biomedical applications, must possess good biocompatibility. To check for toxicity and cell viability, we conducted an LDH and WST-1 cytotoxicity analysis for the NIH3T3 cell line, while it was incubated with composite films. The LDH results (Figure 13a) revealed that the LDH released from the damaged cells was less than 15% in all cases and fell within acceptable levels of toxicity [11]. The one-way ANOVA showed that the addition of pumpkin powder significantly increased the LDH toxicity, with an R^2^ of 0.9236 and a *p*-value of 0.002. The PI films showed an average LDH toxicity of 3.5%, while, with the addition of pumpkin powder, the toxicity increased to an average of 4.9%. The addition of niaouli oil further increased the toxicity in the PIP films and showed a significant difference in the mean data, with an R^2^ of 0.9935 and a *p*-value of 0.00. Though the addition of niaouli oil imparted a slight toxicity to the films, the overall toxicity still fell within the acceptable range [5]. Figure 13b shows the viability of the NIH3T3 cells during the toxicity assay. The results revealed that as the amount of niaouli oil increased, the cell viability decreased. There was no statistical significance between the cell viability data for the PI and PIP films (an R^2^ of 0.2475 and a *p*-value of 0.31). The addition of niaouli oil resulted in a slight decrease in cell viability, which can be seen from the one-way ANOVA values of an R^2^ of 0.9309 and a *p*-value of 0.00). However, the overall viability still remained above 80%, which gave the prepared films a nontoxic status according to the ISO-10993-5 standard [11,74,75,76].

NIH3T3 cell proliferation was analyzed while incubated with the prepared films (Figure 13c). The mitochondrial activity was assessed on day 1, day 3, and day 7. The PI and PIP films showed the highest colorimetric absorbance, in comparison to the negative control (NC), at all-time intervals. The addition of the niaouli Pickering emulsion decreased the NIH3T3 cell proliferation, as can be seen in the toxicity studies, but the proliferation percentage still fell within the accepted levels.

## 4. Conclusions

In the current study, niaouli essential oil-loaded Pickering emulsions were stabilized with β-cyclodextrin. To provide both sustainability and a better antibacterial effect, pumpkin powder was added to the films. SEM micrographs showed that the incorporation of β-CD/niaouli essential oil into the PVA/inulin matrix resulted in the formation of tiny pores on the Pickering film surfaces. Physical interaction between the polymer matrices and β-CD/citronella essential oil inclusion complexes was confirmed by the FT-IR spectrum. There was no significant difference in the thermal properties of the materials. Further, PIP-N3 Pickering films had the highest hydrophobicity, with a 94.2° ± 4.7° value, and the highest EAB, with a 123.8 ± 38.1% value. The presence of niaouli essential oil strengthened and improved the antibacterial activity of the resulting Pickering films. According to the zone inhibition test, the PIP-N5 samples had good antibacterial efficiency against both *E. coli* and *S. aureus*. Unfortunately, pumpkin powder did not show a synergistic effect with niaouli essential oil in the Pickering films. After evaluating the result, it was determined that a β-CD/inclusion complex rate of 1:3 may be the optimum for obtaining a Pickering film.

## Figures and Tables

**Table 1:** The composition of PVA/inulin-based composite/Pickering foams.

Sample ID	PVA (%*w*/*v*)	Pumpkin Powder (%*w*/*v*)	Inulin (%*w*/*v*)	Glycerol (%*v*/*v*)	β-cyclodextrin/Niaouli Essential Oil	MBAm (*w*/*v*)	Citric Acid (*w*/*v*)
PI	10	-	10	4	-	0.1	2.4
PIP	10	2.5	10	4	-	0.1	2.4
PIP-N1	10	2.5	10	4	1:1	0.1	2.4
PIP-N3	10	2.5	10	4	1:3	0.1	2.4
PIP-N5	10	2.5	10	4	1:5	0.1	2.4

**Table 2:** Decomposition temperatures of biocomposite films at 10% (Td_10_), 50% (Td_50_), and 90% (Td_90_) residual weight.

Sample ID	T_d10_	T_d50_	T_d90_	Char Content (%) at 900 °C
PI	195.6	337.9	607.4	1.49
PIP	178.1	333.2	610.9	1.33
PIP-N1	175.6	323.1	605.1	1.13
PIP-N3	176.6	326.6	603.7	0.52
PIP-N5	166.6	317.6	601.6	1.49

**Table 3:** Antibacterial efficiency of the films.

Bacterial Strains	PI	PIP	PIP-N1	PIP-N3	PIP-N5
*Escherichia coli*	-	-	-	12	17
*Staphylococcus aureus*	-	-	-	-	16
*Pseudomonas aeruginosa*	-	-	-	-	-

## Data Availability

The data presented in this study are available on request from the corresponding author.
